# Timescales of Intrinsic BOLD Signal Dynamics and Functional Connectivity in Pharmacologic and Neuropathologic States of Unconsciousness

**DOI:** 10.1523/JNEUROSCI.2545-17.2018

**Published:** 2018-02-28

**Authors:** Zirui Huang, Xiaolin Liu, George A. Mashour, Anthony G. Hudetz

**Affiliations:** ^1^Department of Anesthesiology and Center for Consciousness Science, University of Michigan, Ann Arbor, Michigan 48109 and; ^2^Department of Radiology, Medical College of Wisconsin, Milwaukee, Wisconsin 53226

**Keywords:** anesthesia, consciousness, fMRI, functional connectivity, propofol, temporal receptive window

## Abstract

Environmental events are processed on multiple timescales via hierarchical organization of temporal receptive windows (TRWs) in the brain. The dependence of neural timescales and TRWs on altered states of consciousness is unclear. States of reduced consciousness are marked by a shift toward slowing of neural dynamics (<1 Hz) in EEG/ECoG signals. We hypothesize that such prolongation of intrinsic timescales are also seen in blood-oxygen-level-dependent (BOLD) signals. To test this hypothesis, we measured the timescales of intrinsic BOLD signals using mean frequency (MF) and temporal autocorrelation (AC) in healthy volunteers (*n* = 23; male/female 14/9) during graded sedation with propofol. We further examined the relationship between the intrinsic timescales (local/voxel level) and its regional connectivity (across neighboring voxels; regional homogeneity, ReHo), global (whole-brain level) functional connectivity (GFC), and topographical similarity (Topo). Additional results were obtained from patients undergoing deep general anesthesia (*n* = 12; male/female: 5/7) and in patients with disorders of consciousness (DOC) (*n* = 21; male/female: 14/7). We found that MF, AC, and ReHo increased, whereas GFC and Topo decreased, during propofol sedation. The local alterations occur before changes of distant connectivity. Conversely, all of these parameters decreased in deep anesthesia and in patients with DOC. We conclude that propofol synchronizes local neuronal interactions and prolongs the timescales of intrinsic BOLD signals. These effects may impede communication among distant brain regions. Furthermore, the intrinsic timescales exhibit distinct dynamic signatures in sedation, deep anesthesia, and DOC. These results improve our understanding of the neural mechanisms of unconsciousness in pharmacologic and neuropathologic states.

**SIGNIFICANCE STATEMENT** Information processing in the brain occurs through a hierarchy of temporal receptive windows (TRWs) in multiple timescales. Anesthetic drugs induce a reversible suppression of consciousness and thus offer a unique opportunity to investigate the state dependence of neural timescales. Here, we demonstrate for the first time that sedation with propofol is accompanied by the prolongation of the timescales of intrinsic BOLD signals presumably reflecting enlarged TRWs. We show that this is accomplished by an increase of local and regional signal synchronization, effects that may disrupt information exchange among distant brain regions. Furthermore, we show that the timescales of intrinsic BOLD signals exhibit distinct dynamic signatures in sedation, deep anesthesia, and disorders of consciousness.

## Introduction

The capacity of the brain to integrate complex streams of information requires a multitude of coexisting spatiotemporal scales. In the spatial domain, a basic organizing principle of the visual system is the increasingly large spatial receptive fields of neurons along hierarchical cortical pathways (Hubel, 1988). An analogous gradient of scaling and selectivity also exists in the temporal domain. Recent studies reveal that environmental events are processed on multiple timescales via a hierarchical organization of temporal receptive windows (TRWs) in the brain (Hasson et al., 2008; Lerner et al., 2011; Murray et al., 2014). The TRW of a neuron is defined as the length of time before a response during which sensory information may affect that response. Therefore, the TRWs reflect the timescale on which a specific brain region typically processes information. For example, short TRWs of early sensory areas process rapid sensory information, whereas longer TRWs of higher-order brain areas integrate perceptual and cognitive events that unfold over longer time periods (Hasson et al., 2008; Chaudhuri et al., 2015). Although it has not been demonstrated conclusively, the size of TRWs in different brain regions has been assumed to be proportional to the timescales of intrinsic BOLD signal dynamics (Honey et al., 2012; Stephens et al., 2013).

The hierarchy of neural timescales permits the brain to link multiple perceptual timescales, thus constructing a temporal continuity of conscious experience (Northoff and Huang, 2017). Anesthetic drugs induce a reversible suppression of consciousness and thus offer a unique opportunity to investigate the potential state dependence of neural timescales. States of reduced consciousness are marked by a shift toward slowing of neural dynamics (<1 Hz) in EEG/ECoG signals (Lewis et al., 2012; Ní Mhuircheartaigh et al., 2013). We hypothesize that such prolongation of intrinsic timescales may be also seen in blood-oxygen-level-dependent (BOLD) signals, which may be linked to simultaneous changes in local and global neuronal interactions that support the information integration necessary for consciousness (Alkire et al., 2008; Tononi et al., 2016).

Most previous studies on anesthetic-induced unconsciousness have focused on the spatial aspect of functional alterations such as functional connectivity using both fMRI (Boveroux et al., 2010; Schrouff et al., 2011; Schröter et al., 2012; Guldenmund et al., 2013, 2016; Liu et al., 2013a; Bonhomme et al., 2016) and EEG/ECoG (Lee et al., 2009, 2013; Ku et al., 2011; Boly et al., 2012; Gómez et al., 2013; Jordan et al., 2013; Mashour, 2014). However, the possible relationship between intrinsic functional timescales and the spatial reconfigurations of connectivity during diminished consciousness remains largely unknown and therefore warrants examination. If anesthetic drugs indeed cause a prolongation of intrinsic functional timescales of the brain, which is paralleled by an increase of local neuronal synchronization (Supp et al., 2011), then there are at least two possible mechanisms linking the temporal and spatial features of neural dynamics. The first possibility is that, as anesthetics block distant (long-range) communication, they functionally isolate major brain areas from each other. This isolation may promote the local synchronization of neuronal populations, reflected in the increase in temporal autocorrelation of each disconnected brain region. The second possibility is that anesthetics may exert a direct synchronizing effect on neurons or neuronal networks, increasing local and/or regional functional connectivity, which then impedes distant communication.

To test our hypothesis regarding the prolongation of intrinsic timescales and the postulated two alternative mechanisms, we examined the relationships between the timescales of intrinsic BOLD signals (local/voxel level) and its regional (across neighboring voxels) and global/distant (whole brain level) functional connectivity in experiments performed with resting-state fMRI in healthy volunteers undergoing graded levels of sedation with propofol. Intrinsic timescales were measured by mean frequency (MF) and temporal autocorrelation. Regional, global functional connectivity and topographical similarity were assessed and compared with the timescale indices. Finally, to interpret these experiments in relation to other unconscious states, we extended our analysis to data from participants exposed to surgical levels of general anesthesia and patients with disorders of consciousness (DOC).

## Materials and Methods

### 

#### 

##### Experimental setup and design.

We report findings from three sets of data. The bulk of the report is based on the first dataset (Dataset-1) obtained at the Medical College of Wisconsin (MCW). The Institutional Review Board of MCW approved the experimental protocol. Part of Dataset-1 was previously published using analyses different from those applied here (Liu et al., 2017a, b). In this study, we used data from 23 healthy volunteers (male/female 14/9; 19–35 years). Fifteen subjects received four 15 min scans in wakefulness, propofol-induced light and deep sedation (1–2 μg/ml plasma concentration), and recovery. Transition from deep sedation to recovery (20 min scans) was recorded in 16 subjects, eight of which were part of the 15. Additional data for transition states were obtained from eight other subjects. Two levels of responsiveness were targeted: light sedation, in which volunteers showed lethargic response to verbal commands (observer's assessment of alertness/sedation, OAAS, score 4; Chernik et al., 1990), and deep sedation, during which volunteers showed no response to verbal commands (OAAS score 2-1). The corresponding target plasma concentrations vary across subjects (light sedation: 0.98 ± 0.18 μg/ml; deep sedation: 1.88 ± 0.24 μg/ml) because of the variability in individual sensitivity to anesthetics. At each level of sedation, the plasma concentration of propofol was maintained at equilibrium by continuously adjusting the infusion rate to maintain the balance between accumulation and elimination of the drug. The infusion rate was manually controlled as guided by the output of a computer simulation developed for target-controlled drug infusion (STANPUMP; Shafer, 1996) based on the pharmacokinetic model (Marsh et al., 1991). Transition was initiated at the end of deep sedation by stopping the infusion pump. Eight subjects' transition data were acquired immediately after the onset of infusion stop and 7 subjects' transition data acquisition was started 3 min before the onset of infusion stop. One subject's transition data were acquired starting 10 min before the stop of infusion and an additional 8 min scan was performed after the 20 min scan. To make the data consistent across subjects, we aligned the time of infusion stop and used the maximal data length available across all subjects (17 min). Standard American Society of Anesthesiologists (ASA) monitoring was conducted during the experiment, including electrocardiogram, noninvasive blood pressure cuff, pulse oximetry, and end tidal carbon dioxide gas monitoring. Supplemental oxygen was administered prophylactically via nasal cannula.

##### fMRI data acquisition and preprocessing.

Resting-state fMRI (rs-fMRI) data were acquired at the MCW using a 3 T Signa GE 750 scanner (GE Healthcare) with a standard 32-channel transmit/receive head coil. Functional imaging data were acquired using gradient-echo EPI images of the whole brain (41 slices, TR/TE = 2000/25 ms, slice thickness = 3.5 mm, in-plane resolution = 3.5 × 3.5 mm; FOV = 224 mm, flip angle = 77°, image matrix: 64 × 64). High-resolution spoiled gradient-recalled echo anatomical images were acquired before the functional scans with TE/TR/TI, 8.2/3.2/450 ms; slice thickness, 1 mm; number of slices, 150; flip angle, 12°; field of view, 24 cm; matrix size, 256 × 256.

Preprocessing steps were implemented in AFNI (http://afni.nimh.nih.gov/) including: (1) discarding the first two frames of each fMRI run; (2) physiological noise correction through removal of time-locked cardiac and respiratory artifacts using RETROICOR (Glover et al., 2000); (3) slice timing correction; (4) rigid body correction/realignment within and across runs; (5) coregistration with high-resolution anatomical images; (6) spatial normalization into Talaraich stereotactic space; (7) resampling to 3 × 3 × 3 mm^3^ voxels; (8) regressing out linear and nonlinear drift (equivalent to a high-pass filtering of 0.0067 Hz), head motion and its temporal derivative, and mean time series from the white matter (WM) and CSF to control for non-neural noise (Fox et al., 2005; the WM and CSF masks were eroded by one voxel to minimize partial voluming with gray matter; Chai et al., 2012); (9) spatial smoothing with 8 mm full-width at half-maximum isotropic Gaussian kernel; and (10) the time course per voxel of each run was normalized to zero mean and unit variance (*z*-value), accounting for differences in variance of non-neural origin (e.g., distance from head coil; He, 2011; Stephens et al., 2013).

The issue of head motion artifacts was addressed rigorously based on prior studies (Power et al., 2012, 2014; van Dijk et al., 2012). The estimated motion parameters per subject were inspected for each frame and each sliding window (see below for details of sliding window analysis). Sliding windows that contained head motion larger than 1.5 mm translation or 1.5° rotation (half voxel size; processed data are 3 mm isotropic voxels derived from 3.5 × 3.5 × 3.5 mm acquisition voxels) were censored out from subsequent analysis. The mean proportion of windows censored out was 5.2% in wakefulness, 9.8% in light sedation, 12.2% in deep sedation, 8.7% in transition, and 6.3% in recovery. This censoring was not framewise; it flagged out the entire window (4 min length) if motion in any of the contained frames exceeded threshold. Therefore, this censoring did not damage the temporal continuity of the data within each window. The calculated indices of head motion (shift and rotation) for each subject (Zang et al., 2007) were similar across conditions.

Applying stricter thresholds for framewise displacement (FD) motion censoring; for example, FD > 0.15 mm (Power et al., 2014) would damage the temporal continuity of the data and thus introduce artifacts into the temporal measures including autocorrelation and power spectrum. To confirm that our relatively liberal motion censoring described above did not affect the results, we also sought a stricter threshold that allowed us to preserve >50% subjects (8/15) with >50% continuous data length (7.5 min/15 min) in wakefulness, light sedation, deep sedation, and recovery (excluding transition states). To this end, the FD was first calculated using framewise Euclidean norm (square root of the sum squares) of the six-dimension motion derivatives. We then tested multiple FD thresholds, ranging from stringent (FD > 0.2 mm; Power et al., 2014) to lenient (FD > 0.5 mm; Power et al., 2012) thresholds with 0.1 mm steps. We found that FD > 0.4 mm preserved 9 subjects with >249 frames (8.3 min) continuous data across the 4 conditions. To achieve equal degrees of freedom, we selected equal length of continuous data (249 frames) across the four conditions and nine subjects. We then calculated all measures of interest without sliding window analysis and performed group analyses for confirmation of results. We calculated the mean and SD of Euclidean norm time series (Mean-enorm and SD-enorm, respectively) for each subject and each condition and performed group-level repeated-measures ANOVA. We then tested whether including head motion indices (Mean-enorm and SD-enorm) as covariants in the group analysis affected our results (Yan et al., 2013).

##### Definition of functional networks.

We adopted a well established node template from a previous study (Power et al., 2011) containing 264 putative functional areas (10 mm diameter spheres, 32 voxels per sphere) across the whole-brain gray matter. The original template consisted of 11 functional networks plus 36 uncertain areas (Power et al., 2011; Cole et al., 2014; Huang et al., 2017). In this study, we excluded the cerebellum and uncertain areas and thus included 10 functional networks (226 functional areas in total), namely, subcortical (Sub), dorsal attention (DA), ventral attention (VA), default mode (DMN), frontoparietal task control (FPTC), cingulo-opercular task control (COTC), salience (Sal), sensory/somatomotor (SS), auditory (Audi), and visual networks (Visual). The functional mask of the 10 networks' union was defined as the global mask, which was used to extract the global value for each of the following measurement.

##### Sliding window analysis.

We adopted a dynamic sliding window approach in the following analyses, which has been used previously (Hutchison et al., 2013; Barttfeld et al., 2015; Hudetz et al., 2015). Two considerations motived us to perform this analysis. First, for the transition data, from stopping the pump to recovery, we assumed that the measures (see below) should change (either increase or decrease) as a function of time. The sliding window analysis is an appropriate way to track this dynamic change. To make it consistent across conditions (wakefulness, light sedation, deep sedation, and recovery), this sliding window analysis was applied to the entire dataset. Second, we did not assume that a constant estimated propofol concentration (during light sedation and deep sedation) would necessarily yield a constant anesthetic effect in the brain. This also supports a sliding window analysis.

One concern with the sliding window approach is the choice of window size because it has been varied from tens of seconds to several minutes in previous practices (Hutchison et al., 2013; Barttfeld et al., 2015; Hindriks et al., 2016; Laumann et al., 2017; Tagliazucchi et al., 2016). Smaller window size may capture more transient changes of the dynamics, but it can reduce the statistical reliability and make it more difficult to perform group averaging of dynamic time courses (Hindriks et al., 2016). Conversely, longer window size may capture changes that are more static with larger statistical reliability (e.g., for a given brain state induced by anesthetics). Because the focus of this study was to delineate the state transitions as a function of drug effect, we chose a relatively long window size of 4 min (120 frames) with 20 s (10 frames) time step. Choosing a longer sliding window permitted us to average the time course of a given measurement across subjects. In this sense, the time courses yielded by the longer window size mainly reflect the dynamic changes induced by the anesthetics instead of intrinsic variabilities. Another consideration was that the window size provided the sufficient length of data for calculating the signal's temporal autocorrelation and power spectrum. Finally, the commonly used frequency range for analyzing the fluctuations of spontaneous brain activity has been between 0.01 and 0.1 Hz (Fox and Raichle, 2007). Because 4 min data may not provide an accurate estimation of the lowest frequencies near 0.01 Hz (with only two cycles in the 4 min's data), we made a trade-off by band-pass filtering the BOLD signal in 0.02–0.1 Hz and normalized to zero mean and unit variance for each sliding window.

Mean Frequency. The power spectrum was obtained by computing the periodogram of each voxel time series using AFNI program 3dPeriodogram. Tapering was applied using the Hamming window to reduce bias and error variance for spectral estimation in finite data (Babadi and Brown, 2014). The MF of a spectrum was calculated as the sum of the product of the spectrogram power intensity and the frequency, divided by the total sum of spectrogram power intensity as follows:

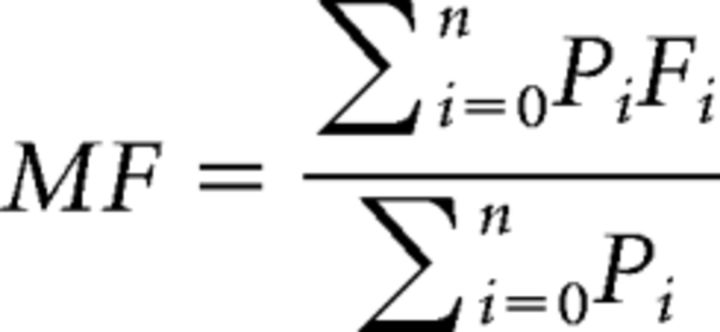
 Where *n* is the number of frequency bins in the spectrum, *F*_i_ is the frequency of spectrum at bin *i* of *n*, and *P*_i_ is the power intensity of spectrum at bin *i* of *n*. Lower MF indicates a shift toward a longer timescale or slower dynamics. The MF was calculated for each sliding window and each voxel in the brain, yielding a time course of 3D MF maps. The MF time courses were then extracted from the global brain mask and different networks for secondary group analysis.

##### Temporal autocorrelation.

Temporal autocorrelation of the intrinsic BOLD signals was first calculated at lag-1 (first-order) as lag-1 temporal autocorrelation (AC1) = corr(*y*_t_, *y*_t−1_), where *y* is the fMRI-BOLD time course. Lag-1 autocorrelation is a robust measure of the decay of the autocorrelation function that is commonly used to characterize dynamical systems including neurophysiological and fMRI data (Scheffer et al., 2009, 2012; Kaneoke et al., 2012; Tagliazucchi et al., 2016; Meisel et al., 2017a, b). A higher AC1 indicates a shift toward slower dynamics. For a more comprehensive characterization of BOLD timescales, we also examined the autocorrelation function from 2 to 50 s (2 s step, given TR = 2 s) yielding 25 correlation coefficients. The 50 s cutoff matched the upper limit of the filtered fMRI frequency band (0.02–0.1 Hz). We observed two time ranges of interest, 10–18 s and 30–38 s, which showed a clear difference across conditions (see Fig. 3*A* for visualization). The latter range included the previously reported 34 s time lag of interest that showed the strongest relationship between structural topology and spontaneous activity (Sethi et al., 2017). We defined ACR1 and ACR2 by the sum of the absolute value of correlation coefficients within the two frequency ranges, respectively. AC1, ACR1, and ACR2 were calculated for each sliding window and each voxel in the brain, yielding respective 3D time course maps. Similar to the analysis pipeline for MF, autocorrelation time courses were extracted from the global brain mask and different networks for secondary group analysis.

##### Regional functional connectivity.

Regional homogeneity (ReHo) was calculated at the voxel level using Kendall's coefficient of concordance between the BOLD time series for specified voxel and those of its 26 nearest neighbors (∼2 mm radius sphere; Zang et al., 2004). ReHo quantifies the intraregional signal correlation. ReHo analysis was performed for each sliding window with the AFNI program 3dReHo, which yields a voxelwise ReHo map (Fisher's *Z* transformed). Because spatial smoothing could artificially enhance ReHo and reduce its reliability (Zuo et al., 2013), we calculated ReHo from nonsmoothed BOLD time series. Spatial smoothing was subsequently applied with an 8 mm full-width at half-maximum Gaussian kernel to the ReHo maps.

##### Distant functional connectivity.

Functional connectivity in the centimeter range was calculated based on the aforementioned node template, including 10 networks with 226 functional areas (Power et al., 2011), where the minimal Euclidian distance between 2 centers of any pair of nodes is 2 cm. Distant connectivity is defined over the range >1.4 cm (Sepulcre et al., 2010). This is different from ReHo, which reflects connectivity within ∼2 mm radius sphere. Next, we computed the Pearson correlation coefficient of the time courses between each pair of nodes, yielding a pairwise 226 × 226 correlation matrix (Fisher's *Z* transformed) per sliding window. For a measure of global functional connectivity (GFC), we calculated the average of the triangular of the matrix of size 226 × (226 − 1)/2.

##### Topographical similarity.

Topographical similarity (Topo) was defined by the correlation coefficient between a reference correlation matrix (triangular) and a correlation matrix for each sliding window of each subject. The reference correlation matrix was obtained by averaging the correlation matrix of 15 min rs-fMRI during wakefulness across 15 subjects. Therefore, Topo quantifies the divergence of spatial connectivity configuration (among the nodes) from baseline over time (see Tagliazucchi et al., 2016 for a similar measurement of similarity between functional and anatomical topography).

##### Deep general anesthesia and patients with DOC.

For a comparison with deeper states of unconsciousness, the above analyses (except for the sliding window analysis due to limited data length) were extended to two other independent datasets. Dataset-2 included 12 subjects (male/female: 5/7; age: 32–63 years) with three 8 min rs-fMRI scans from wakefulness, light sedation at 1.3 μg/ml, and deep anesthesia at 4 μg/ml propofol. This received approval from the Ethics Committee of Huashan Hospital, Fudan University. All subjects gave written informed consent. The subjects were selected on elective trans-sphenoidal approach to control on pituitary microadenoma (<10 mm in diameter without sella expansion using radiological and plasma endocrinal indicator). Subjects were ASA physical status I or II grade with no history of craniotomy, cerebral neuropathy, or vital organ dysfunction. All subjects received intravenous propofol anesthesia by target-controlled infusion. This study used a Siemens 3T MAGNETOM scanner with a head coil to extract whole-brain gradient-echo EPI images (slice number = 33, TR/TE = 2000/30 ms, slice thickness = 5 mm, field of view = 210 mm, flip angle = 90°, image matrix: 64 × 64). High-resolution anatomical images were obtained. Another 23 healthy subjects provided control data (no propofol infusion) with the same scanning protocol. The latter dataset was used to calculate the reference correlation matrix for topographical similarity calculation in both Dataset-2 and the following Dataset-3.

Dataset-3 included 21 patients (male/female: 14/7) with DOC and 28 healthy control subjects (male/female: 14/14). This dataset derives from our previous published data (Huang et al., 2014, 2016). Because brain lesions may affect topographical similarity, for this analysis, we included only 12 DOC subjects who had well preserved anatomical structures (see Huang et al., 2016 for the inclusion and exclusion criteria).

##### Statistical analysis.

In Dataset-1, the mean (across windows) of each measured quantity (MF, AC1, ACR1, ACR2, ReHo, GFC, and Topo) for a given state of consciousness (e.g., light sedation) was tested against the wakeful baseline (paired-sample *t* test; except for transition with two-sample *t* test). The slope of each measurement (as a function of time; fraction time unit in percentage of windows) of each state was tested against zero (one-sample *t* test). In Dataset-2, the mean of each measurement for a given condition was tested against the wakefulness baseline by paired-sample *t* test. In Dataset-3, the mean of each measurement for DOC group was tested against the healthy control group by two-sample *t* test. Unless otherwise stated (e.g., uncorrected), *p*-values with FDR correction were reported.

## Results

### Decrease of MF during sedation

**Figure 1.**
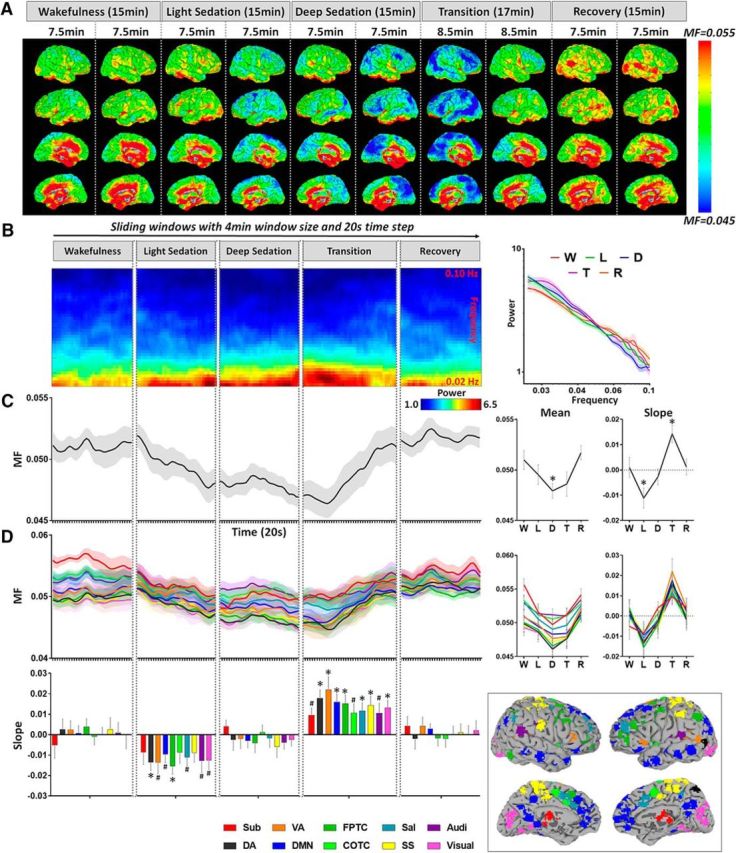
MF of BOLD signal during five conditions: wakefulness (W), light sedation (L), deep sedation (D), transition (T), and recovery (R). ***A***, MF was calculated for each voxel across the whole brain. Each subject's data were split into two halves for each condition and then averaged across subjects for illustrative purposes. ***B***, Group-level sliding window (window length, 4 min; time step, 20 s) power spectra of the whole brain (left) and mean power spectra (log-log plots) of different conditions (right). The power spectra were smoothed with a Hamming window of five neighboring frequency bins for illustrative purposes. ***C***, Group-level global MF time course was obtained by averaging the MF values across all voxels in the global brain mask for each sliding window and concatenating across the sliding windows. The mean of MF (across windows) for a given condition (e.g., light sedation) was tested against the wakefulness baseline. The slope of MF versus time for each condition was tested against zero. ***D***, MF time course, mean, and slope of MF for each condition were extracted from 10 predefined brain networks. These networks include subcortical (Sub), dorsal attention (DA), ventral attention (VA), default mode (DMN), frontoparietal task control (FPTC), cinguloopercular task control (COTC), salience (Sal), sensory/somatomotor (SS), auditory (Audi), and visual networks (Visual) (Power et al., 2011). Shaded areas and error bars indicate ± SEM. *FDR corrected *p* < 0.05; #uncorrected *p* < 0.05.

We observed a graded global decrease of MF from wakefulness to light and deep sedation, followed by an increase during transition and return to baseline in recovery (Fig. 1*A–C*). A significant mean difference of global MF between wakefulness and deep sedation was found (*p* = 0.038). We also measured the slope of MF versus time for each state. A negative slope was seen during light sedation (*p* = 0.029) and a positive slope was seen during the transition period (*p* = 0.004). Significant slopes indicated that, despite the presumably pharmacokinetically stable propofol plasma concentration during light or deep sedation, the reconfiguration of the power spectrum (and MF) continued to progress with time. The MF (∼0.05 Hz) obtained during wakefulness was consistent with previous fMRI and computation modeling studies (Glerean et al., 2012; Ponce-Alvarez et al., 2015) in which 0.04–0.07 Hz in fMRI contains more robust and functionally relevant signals than the other bands.

To determine the possible difference in the contribution of specific networks, we examined the MF values for predefined functional networks. We found that the MF changes of all networks behaved similarly across different states (Fig. 1*D*).

### Increase of temporal autocorrelation during sedation

**Figure 2.**
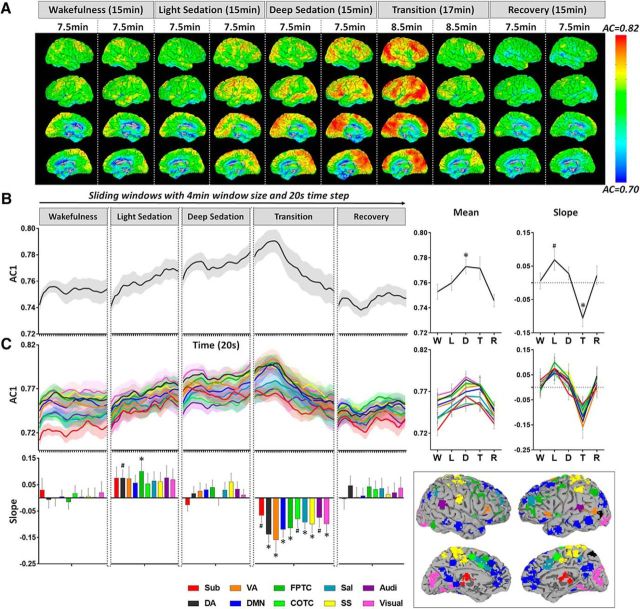
Lag-1 temporal autocorrelation coefficient (AC1) of resting-state fMRI signals during five conditions, wakefulness (W), light sedation (L), deep sedation (D), transition (T), and recovery (R). ***A***, AC1 was calculated for each voxel across the whole brain. Each subject's data were split into two halves for each condition and then averaged across subjects for illustrative purposes. ***B***, Group-level global AC1 time course obtained by averaging the AC1 values across all voxels in the global brain mask for each sliding window and concatenating cross the sliding windows (window length, 4 min; time step, 20 s). The mean of AC1 (across windows) for a given condition (e.g., light sedation) was tested against the wakefulness baseline. The slope of AC1 versus time for each condition was tested against zero. ***C***, AC1 time course, mean and slope of AC1 for each condition were extracted from 10 predefined brain networks. These networks include subcortical (Sub), dorsal attention (DA), ventral attention (VA), default mode (DMN), frontoparietal task control (FPTC), cinguloopercular task control (COTC), salience (Sal), sensory/somatomotor (SS), auditory (Audi), and visual networks (Visual) (Power et al., 2011). Shaded areas and error bars indicate ± SEM. *FDR corrected *p* < 0.05; #uncorrected *p* < 0.05.

The timescale of intrinsic BOLD signal was also characterized by the temporal autocorrelation. Based on the observed changes in MF, we expected to see an increase of AC1 during sedation. As anticipated, we observed a graded global increase of AC1 from wakefulness to light and deep sedation, followed by a decrease during transition and return to baseline in recovery (Fig. 2*A*,*B*). A significant mean difference of global AC1 between wakefulness and deep sedation was found (*p* = 0.042). A positive slope of AC1 was seen during light sedation (*p* = 0.038, uncorrected) and a negative slope of AC1 was seen during the transition period (*p* = 0.004). The AC1 changes of all networks behaved similarly across different states (Fig. 2*C*).

**Figure 3.**
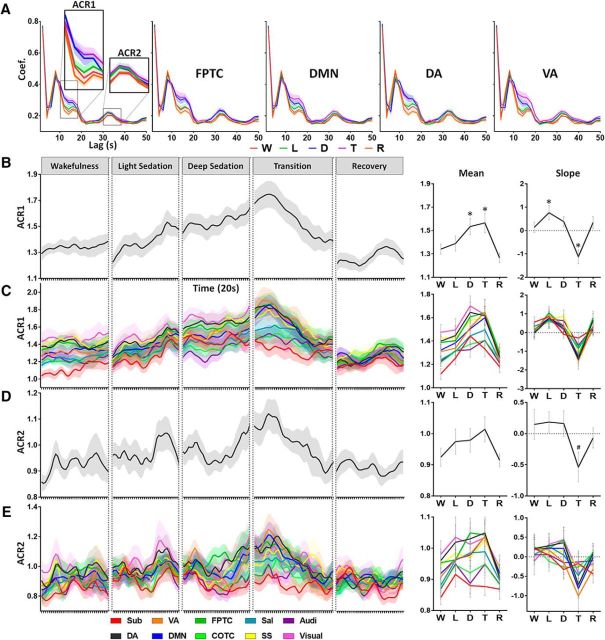
Temporal autocorrelation of resting-state fMRI signals during five conditions, wakefulness (W), light sedation (L), deep sedation (D), transition (T), and recovery (R). ***A***, Temporal autocorrelation (absolute coefficients) with multiple time lags (2–50 s) was calculated for each voxel and then averaged across all voxels in the global brain mask (top left), as well as four representative networks. The ACR1 and ACR2 were defined by summing the absolute coefficient within the two time ranges of interest, 10–18 s and 30–38 s, respectively. ***B***, Group-level global ACR1 time course (window length, 4 min; time step, 20 s). The mean of ACR1 (across windows) for a given condition (e.g., light sedation) was tested against the wakefulness baseline. The slope of ACR1 versus time for each condition was tested against zero. ***C***, ACR1 for 10 predefined brain networks. Same illustration applies to ACR2 in ***D*** and ***E***. Networks include subcortical (Sub), dorsal attention (DA), ventral attention (VA), default mode (DMN), frontoparietal task control (FPTC), cinguloopercular task control (COTC), salience (Sal), sensory/somatomotor (SS), auditory (Audi), and visual networks (Visual) (Power et al., 2011). Shaded areas and error bars indicate ± SEM. *FDR corrected *p* < 0.05; #uncorrected *p* < 0.05.

In addition to lag-1 temporal autocorrelation, we found two other time ranges (lags), 10–18 s (ACR1) and 30–38 s (ACR2), which showed differences across conditions (Fig. 3*A*). We observed similar results of ACR1 and ACR2 to those in AC1 (Fig. 3*B–E*), albeit the changes were smaller in ACR2. Significant mean differences of global ACR1 between wakefulness and deep sedation (*p* = 0.045) as well as between wakefulness and transition (*p* = 0.047) were found. A positive slope of ACR1 was seen during light sedation (*p* = 0.049) and a negative slope of AC1 was seen during transition (*p* = 0.007). Last, a negative slope of ACR2 was seen during transition (*p* = 0.033, uncorrected).

We demonstrated a global decrease of MF and increase of temporal autocorrelation of intrinsic BOLD signals during sedation, suggesting a prolongation of the brain's intrinsic functional timescales.

### Local, regional, and global functional connectivity during sedation

**Figure 4.**
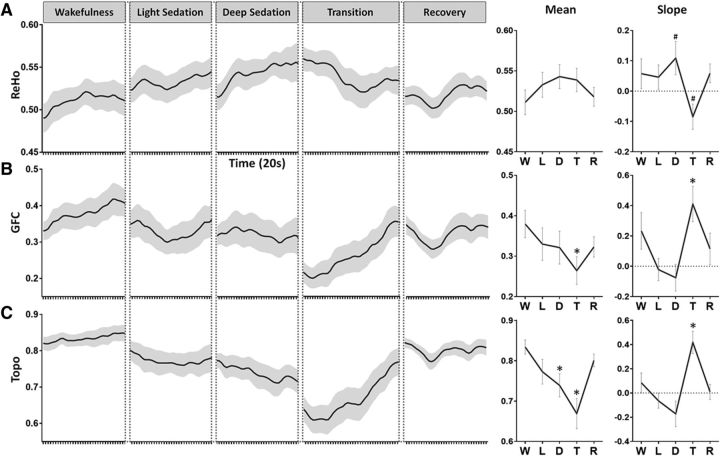
ReHo, GFC, and Topo during five conditions: wakefulness (W), light sedation (L), deep sedation (D), transition (T) and recovery (R). ***A***, Group-level global ReHo time course was obtained by averaging the ReHo values across all voxels in the global brain mask for each sliding window and concatenating cross the sliding windows (window length, 4 min; time step, 20 s). The mean of ReHo (across windows) for a given condition (e.g., light sedation) was tested against the wakefulness baseline. The slope of ReHo versus time for each condition was tested against zero. Same illustration applies to the following measures. ***B***, Group-level GFC time course, mean, and slope of GFC. ***C***, Group-level Topo time course and mean and slope of Topo. Shaded areas and error bars indicate ± SEM. *FDR corrected *p* < 0.05; #uncorrected *p* < 0.05.

A prolongation of temporal autocorrelation of intrinsic BOLD signals at the single-voxel level may reflect an increase of local neuronal synchronization (Supp et al., 2011). Although direct assessment of neuronal synchronization within a single voxel cannot be determined by fMRI, one would expect that local neuronal synchronization may extend to an intermediate spatial scale, such as regional brain areas with tens of voxels as measured by ReHo. Indeed, we observed that the global ReHo changes shared a similar trend with AC1, ACR1, and ACR2. That is, ReHo steadily increased from wakefulness to light/deep sedation, decreased during transition, and returned to baseline during recovery (Fig. 4*A*). We found a positive slope of ReHo during deep sedation (*p* = 0.044, uncorrected) and a negative slope during the transition period (*p* = 0.049, uncorrected). We did not find a significant treatment effect of the global ReHo (mean) across different states by repeated-measures ANOVA. However, when examining ReHo in different networks, significant treatment effects were seen in the subcortical (*F* = 5.44, df = 3, *p* = 0.003), ventral attention (*F* = 2.93, df = 3, *p* = 0.045), and salience (*F* = 3.18, df = 3, *p* = 0.034) networks. This suggests that propofol sedation induced network-specific alterations in regional connectivity

In contrast to ReHo, GFC showed the opposite pattern (Fig. 4*B*). A significant decrease in GFC was found in transition compared with wakefulness baseline (*p* = 0.019) and a positive slope of GFC was seen during transition (*p* = 0.012). The changes of GFC in different networks were indistinguishable from each other and were similar to the global changes.

The changes of Topo showed a similar pattern to that of GFC (Fig. 4*C*). Specifically, significant decreases in Topo were found during deep sedation (*p* = 0.017) and transition (*p* = 0.001) compared with the wakeful baseline. A positive slope of Topo was seen during transition (*p* = 0.002). Collectively, the results of GFC and Topo indicate a breakdown of distant (long-range) functional connectivity and spatial configuration during sedation.

It is important to note that the local measures (MF, AC1, and ACR1) changed during light sedation (significant slopes), whereas global measures (GFC and Topo) did not (no significant slopes). The changes of GFC and Topo occurred mostly during deep sedation and transition rather than light sedation. These results suggest that, during graded sedation, local changes presumably reflecting local neuronal synchronization occur before global changes affecting distant neuronal communication.

**Figure 5.**
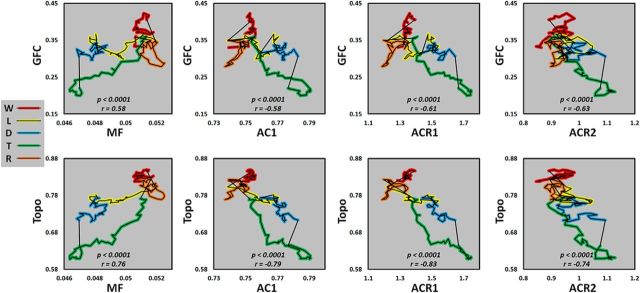
Temporal interdependence of different variables. Trajectory plots are shown at the group level for the changes of GFC and Topo as a function of MF, AC1, sum of absolute autocorrelation coefficient in time range 10–18 s (ACR1), and time range 30–38 s (ACR2). Wakefulness (W), light sedation (L), deep sedation (D), transition (T), and recovery (R).

To illustrate the temporal interdependence of these variables, the trajectory of the changes of global measures (GFC and Topo) were plotted as a function of local measures (MF, AC1, ACR1, and ACR2; Fig. 5). Overall, we observed a positive relationship between global measures and MF and a negative relationship between global measures and temporal autocorrelations (AC1, ACR1, and ACR2).

### Confirmation of results after framewise motion censoring

**Figure 6.**
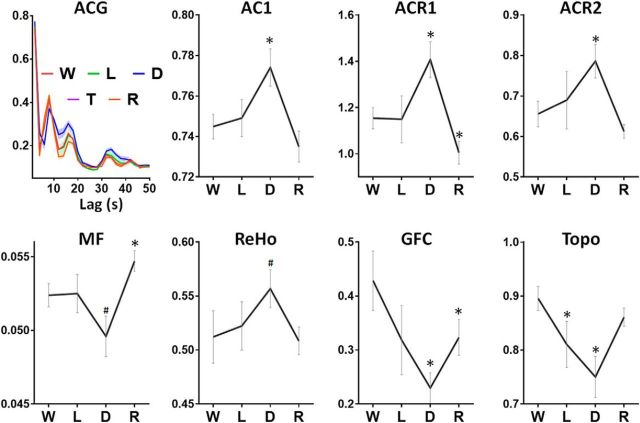
Confirmative results in a subgroup of subject (*n* = 9) by applying more rigorous FD motion censoring in wakefulness (W), light sedation (L), deep sedation (D) and recovery (R). Each measure for a given condition was tested against the wakefulness baseline. Error bars indicate ± SEM. *FDR corrected *p* < 0.05; #uncorrected *p* < 0.05.

To confirm that our results were unaffected by relatively liberal motion censoring, we performed FD motion censoring in a subject subgroup (*n* = 9; 249 frames). The results replicated our main findings with window-wise censoring approach. Specifically, significant differences between wakefulness and deep sedation were found in all measures: AC1 (*p* = 0.012), ACR1 (*p* = 0.019), ACR2 (*p* = 0.048), MF (*p* = 0.043, uncorrected), ReHo (*p* = 0.046, uncorrected), GFC (*p* = 0.008), and Topo (*p* = 0.008). We also found significant differences between wakefulness and light sedation in Topo (*p* = 0.048) and between wakefulness and recovery in ACR1 (*p* = 0.018), MF (*p* = 0.021), and GFC (*p* = 0.031; Fig. 6). In addition, we did not observe any difference between conditions in either Mean-enorm (*F* = 0.26, *p* = 0.85, df = 3) or SD-enorm (*F* = 0.05, *p* = 0.98, df = 3) of the head motion indices using repeated-measures ANOVA. Last, we confirmed that all results remained significant by including the Mean-enorm and SD-enorm as covariance. Together, our findings were robust with different head motion censoring approaches (window-wise and framewise).

### Local, regional, and global alterations in deep general anesthesia and DOC

**Figure 7.**
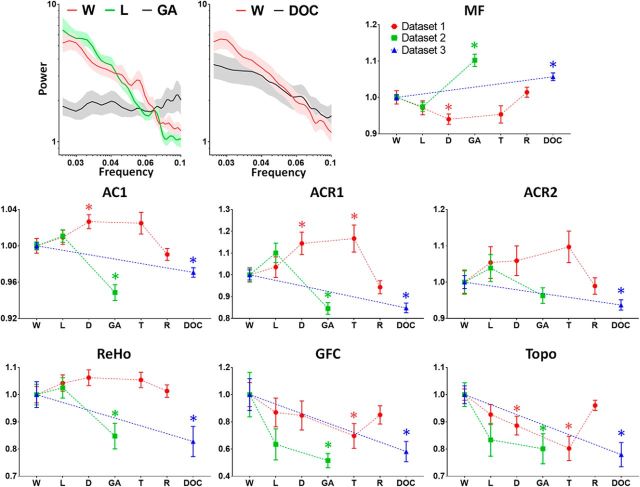
Comparison of corresponding measurements across three datasets. Dataset-1 (main results shown in Figs. 1, 2, 3, and 4) includes 23 healthy volunteers during wakefulness (W), light sedation (L), deep sedation (D), transition (T), and recovery (R). Dataset-2 includes 12 subjects during W, L, and surgical-level general anesthesia (GA). Dataset-3 includes 21 patients with DOC and 28 healthy control subjects (W). The mean of each measurement for a given condition, for example, L (or DOC), was tested against the wakefulness baseline (or healthy controls). For illustrative purposes, each dataset was normalized by dividing the mean of its own baseline (W) because different scanners and data acquisition parameters affect the absolute value of these measurements. Error bars indicate ± SEM. *p < 0.05.

We investigated whether the spatiotemporal alterations of local dynamics and functional connectivity (regional and global) observed in sedation could be seen during a surgical level of deep general anesthesia (Dataset-2) and in DOC patients (Dataset-3). We found that all measures obtained during light sedation in Dataset-2 were consistent with those in Dataset-1. However, there was a significant increase of MF (*p* < 0.001) and a significant decrease of AC1 (*p* < 0.001), ACR1 (*p* = 0.007), and ReHo (*p* = 0.014) during deep general anesthesia in Dataset-2. The other two global measures, GFC and Topo, both showed a significant decrease (*p* = 0.012 for GFC; *p* = 0.007 for Topo) during deep general anesthesia (Fig. 7, green values). Furthermore, the results of ACR1 (*p* < 0.001), ACR2 (*p* = 0.01), ReHo (*p* = 0.019), GFC (*p* = 0.006), and Topo (*p* < 0.001) in DOC patients (vs healthy control group) in Dataset-3 were similar to those during deep general anesthesia in Dataset-2, whereas the MF (*p* < 0.001) and AC1 (*p* < 0.001) values for DOC were between those in wakefulness and deep general anesthesia (Fig. 7, blue values). Collectively, these findings suggest that spatiotemporal alterations of local dynamics and functional connectivity are distinct in sedation, deep general anesthesia, and DOC.

## Discussion

We demonstrated a prolongation of the timescales of intrinsic BOLD signal dynamics during propofol sedation with a decrease of MF and an increase of temporal autocorrelation (AC1, ACR1, and ACR2). These local changes occurred in association with an elevated ReHo, a breakdown of GFC, and a departure from the presumably optimized spatial configuration of the wakeful baseline (Topo). The local changes seemed to occur before global functional alterations, suggesting that propofol promotes local neurons to synchronize, which in turn reconfigures whole-brain connectivity. Furthermore, we showed that deep general anesthesia and DOC could be distinguished from the sedated state by the opposite direction of changes of timescale indices.

### Prolongation of the brain's intrinsic functional timescales during sedation

The global prolongation of the timescales of intrinsic BOLD signals may indicate an enlarged TRW, acting as a low-pass filter or sparse sampling of the inputs from extrinsic and intrinsic sources, reducing the bandwidth of information processing. This is supported by our behavioral assessment that light sedation was associated with lethargic response to verbal commands, whereas deep sedation was associated with absent response to verbal commands. The mismatch between the brain's intrinsic functional timescales and the timescales of experienced world events may not only affect information acquisition at relatively short timescales, but may also impede information integration over longer timescales. This may best be interpreted as information “received but not perceived” (Hudetz, 2006), which may also explain disrupted semantic processing (Davis et al., 2007; Adapa et al., 2014) and auditory predictive coding (Uhrig et al., 2016) during sedation.

### Differential alterations of local, regional, and global functional organizations during sedation

The increase of local temporal autocorrelation may indicate an increase of local neuronal synchronization (Supp et al., 2011; Honey et al., 2012). This is consistent with previous EEG/ECoG studies that found an increase of local phase coherence in alpha-, beta-, and delta-frequency bands during loss of consciousness (Supp et al., 2011; Li et al., 2013; Purdon et al., 2013; Ishizawa et al., 2016; Wollstadt et al., 2017). This was also seen in the neuronal activity of layer 2/3 using two-photon calcium activity imaging (Lissek et al., 2016). Moreover, a recent study provides a link between neuronal timescales (spiking activity) and large-scale signals measured by EEG and fMRI (Meisel et al., 2017a, b). Nevertheless, it is important to recognize that different rhythms seen in EEG/ECoG signals have distinct functions and the exact relationship between fMRI slowing and EEG/ECoG slowing during sedation requires further investigation.

The increase of local neuronal synchronization was also seen at the intermediate spatial scale through increases in the ReHo across neighboring voxels during sedation. This is consistent with a recent rodent study that found strengthened ReHo in several brain areas under six different anesthetic regimens (Wu et al., 2017). The increase of ReHo may be related to an increase of local self-inhibitory connectivity resulting from the enhancement of GABA_A_ receptor activity by propofol (Gómez et al., 2013), particularly the increasing strength and prolonged decay time of inhibitory signaling from interneurons to pyramidal cells and from thalamic reticular neurons to thalamic relay cells (Huguenard and McCormick, 2007; Franks, 2008; Ching et al., 2010).

In contrast to the increase of local/regional signal synchronization, we observed a global reduction of distant functional connectivity during sedation, which is consistent with previous studies (Boveroux et al., 2010; Martuzzi et al., 2010; Schrouff et al., 2011; Schröter et al., 2012; Guldenmund et al., 2013, 2016; Liu et al., 2013a; Bonhomme et al., 2016; Ranft et al., 2016). We also found reduced topographical similarity during sedation, suggesting that the spatial configuration of functional connectivity diverges from that of baseline over time.

Importantly, our results showed that local temporal dynamics change earlier than global functional connectivity and topographical changes. Although the common view is that anesthetics block distant communication (Boveroux et al., 2010; Bonhomme et al., 2016; Hudetz, 2012; Lee et al., 2013), our present findings suggest that the primary effect of anesthetics is the synchronization of neurons in local circuits, increasing local and/or regional functional connectivity. This in turn impairs the ability of local areas to functionally couple with distant ones, reducing the brain's ability to integrate information. This conclusion should be considered tentative. Admittedly, a firm causal relationship between the described events is difficult to assert from our data because of the poor temporal resolution of fMRI and the lack of direct neuronal evidence. Future studies including those with different or noncanonical anesthetics such as ketamine combining fMRI with EEG/ECoG and computational modeling may help to solidify our conclusion.

Despite the presumed equilibrium of propofol plasma concentrations during light and deep sedation, local/regional measures continued to change as indicated by a slope significantly different from zero. These changes suggest a dynamic, progressive reconfiguration of intrinsic brain activity during light and deep sedation, which is unlikely to be a pharmacokinetic effect.

### Deep general anesthesia and DOC have distinct spatiotemporal properties compared with sedation

**Figure 8.**
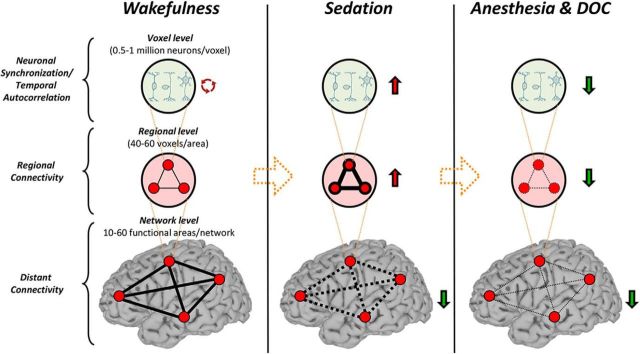
Schematic summary of the consecutive stages of functional alterations during propofol sedation, deep general anesthesia, and DOC. Relative to the control state (wakefulness, left), stage 1 (light to moderate sedation, middle) is marked by an increase of local/regional signal synchrony and consequent breakdown of global connectivity. Stage 2 (deep general anesthesia or DOC, right) collapses both local/regional synchrony and global connectivity.

We examined the spatiotemporal properties at a deeper level of propofol anesthesia and in patients with DOC. Unlike in sedation, there was an increase of MF and a decrease of temporal autocorrelation during deep general anesthesia and DOC. These results suggest that the timescales of intrinsic BOLD signals become globally shorter during deep general anesthesia and DOC. What is the reason for this biphasic behavior of the intrinsic timescales? Why are they longer during sedation but shorter during deep general anesthesia and DOC compared with wakefulness and recovery/emergence? We speculate that this biphasic (or perhaps even multiphasic as seen in our previous animal study; Liu et al., 2013b) phenomenon results from two consecutive stages of functional alterations (Fig. 8). First, an increase of local/regional synchrony breaks down global connectivity during light to moderate sedation and, second, both local/regional synchrony and global connectivity collapse at a high, surgical dose. The two stages of functional alterations may be associated with distinct behavioral signatures of anesthetic sedation and deep general anesthesia. During sedation, subjects can respond purposefully, albeit in a delayed manner, to verbal commands or to tactile stimulation (Brown et al., 2011). This suggests that the sedated brain may still preserve a certain capacity for global information integration (partially preserved long-range functional connectivity), whereas the processing of especially complex contents becomes slow and inefficient (increase of temporal autocorrelation). In contrast, during deep general anesthesia, the subjects are no longer arousable, even by painful stimulation, indicating a profound collapse of local, regional, and long-range functional interactions.

Last, it is noteworthy that the MF and AC1 values in DOC patients were between those in wakefulness and deep general anesthesia, whereas the values for ReHo, GFC, and Topo in DOC patients were indistinguishable from those during deep general anesthesia (Fig. 7). If we assume that some of the DOC patients retained a certain level of residual consciousness (based on the imprecision of clinically differentiating unresponsive wakefulness syndrome from a minimally conscious state) or were at least in a state of wakefulness (Laureys and Schiff, 2012; Kondziella et al., 2016) whereas the patients under deep general anesthesia were not, our results suggest that the state of altered consciousness correlates more closely with the temporal dynamics than with the functional connectivity of brain regions.

### Limitations

Several limitations of our study need to be considered. First, the assessment of the timescales of intrinsic BOLD signals in relation to the TRWs (defined by cognitive tasks) representing the temporal dimension of perception was indirect. The effect of the observed prolongation of intrinsic timescales on behavior, perception, and phenomenology during sedation remains to be established; for example, whether the subject's temporal perception is altered or their “stream of consciousness” is fragmented. Second, despite experimental validations of fMRI as a suitable method to probe the hierarchy of TRWs (Hasson et al., 2008; Lerner et al., 2011; Stephens et al., 2013), the temporal resolution of the fMRI-BOLD signal is admittedly coarse to quantify the entire spectrum of TRWs, particularly at the millisecond to subsecond timescales that are nevertheless associated with important steps of sensory processing. Third, sedation and deep general anesthesia were studied in separate experiments. Therefore, we cannot determine whether the biphasic behavior of intrinsic timescales was in part related to the onset of anesthesia/speed of induction, although this is unlikely given the equilibration time allowed before each scan. Last, it remains to be determined whether our present results can be generalized to other classes of anesthetics, including halogenated ethers such as sevoflurane (amenable to study with fMRI) or non-GABAergic drugs such as ketamine.

### Conclusions

We demonstrate for the first time that sedation with propofol synchronizes local neuronal interactions and prolongs the timescales of intrinsic BOLD signals. This in turn disrupts information exchange among distant brain regions. The intrinsic timescales have distinct neural dynamic signatures in sedation, deep general anesthesia, and DOC. These results improve our understanding of the neural mechanisms of unconsciousness in pharmacologic and neuropathologic states.

## References

[B1] Adapa RM, Davis MH, Stamatakis EA, Absalom AR, Menon DK (2014) Neural correlates of successful semantic processing during propofol sedation. Hum Brain Mapp 35:2935–2949. 10.1002/hbm.22375 24142410 PMC6869007

[B2] Alkire MT, Hudetz AG, Tononi G (2008) Consciousness and anesthesia. Science 322:876–880. 10.1126/science.1149213 18988836 PMC2743249

[B3] Babadi B, Brown EN (2014) A review of multitaper spectral analysis. IEEE Trans Biomed Eng 61:1555–1564. 10.1109/TBME.2014.2311996 24759284

[B4] Barttfeld P, Uhrig L, Sitt JD, Sigman M, Jarraya B, Dehaene S (2015) Signature of consciousness in the dynamics of resting-state brain activity. Proc Natl Acad Sci U S A 112:887–892. 10.1073/pnas.1418031112 25561541 PMC4311826

[B5] Boly M, Moran R, Murphy M, Boveroux P, Bruno MA, Noirhomme Q, Ledoux D, Bonhomme V, Brichant JF, Tononi G, Laureys S, Friston K (2012) Connectivity changes underlying spectral EEG changes during propofol-induced loss of consciousness. J Neurosci 32:7082–7090. 10.1523/JNEUROSCI.3769-11.2012 22593076 PMC3366913

[B6] Bonhomme V, Vanhaudenhuyse A, Demertzi A, Bruno MA, Jaquet O, Bahri MA, Plenevaux A, Boly M, Boveroux P, Soddu A, Brichant JF, Maquet P, Laureys S (2016) Resting-state network-specific breakdown of functional connectivity during ketamine alteration of consciousness in volunteers. Anesthesiology 125:873–888. 10.1097/ALN.0000000000001275 27496657

[B7] Boveroux P, Vanhaudenhuyse A, Bruno MA, Noirhomme Q, Lauwick S, Luxen A, Degueldre C, Plenevaux A, Schnakers C, Phillips C, Brichant JF, Bonhomme V, Maquet P, Greicius MD, Laureys S, Boly M (2010) Breakdown of within- and between-network resting state functional magnetic resonance imaging connectivity during propofol-induced loss of consciousness. Anesthesiology 113:1038–1053. 10.1097/ALN.0b013e3181f697f5 20885292

[B8] Brown EN, Purdon PL, Van Dort CJ (2011) General anesthesia and altered states of arousal: a systems neuroscience analysis. Annu Rev Neurosci 34:601–628. 10.1146/annurev-neuro-060909-153200 21513454 PMC3390788

[B9] Chai XJ, Castañón AN, Ongür D, Whitfield-Gabrieli S (2012) Anticorrelations in resting state networks without global signal regression. Neuroimage 59:1420–1428. 10.1016/j.neuroimage.2011.08.048 21889994 PMC3230748

[B10] Chaudhuri R, Knoblauch K, Gariel MA, Kennedy H, Wang XJ (2015) A large-scale circuit mechanism for hierarchical dynamical processing in the primate cortex. Neuron 88:419–431. 10.1016/j.neuron.2015.09.008 26439530 PMC4630024

[B11] Chernik DA, Gillings D, Laine H, Hendler J, Silver JM, Davidson AB, Schwam EM, Siegel JL (1990) Validity and reliability of the Observer's assessment of Alertness/Sedation scale: study with intravenous midazolam. J Clin Psychopharmacol 10:244–251. 2286697

[B12] Ching S, Cimenser A, Purdon PL, Brown EN, Kopell NJ (2010) Thalamocortical model for a propofol-induced rhythm associated with loss of consciousness. Proc Natl Acad Sci U S A 107:22665–22670. 10.1073/pnas.1017069108 21149695 PMC3012501

[B13] Cole MW, Bassett DS, Power JD, Braver TS, Petersen SE (2014) Intrinsic and task-evoked network architectures of the human brain. Neuron 83:238–251. 10.1016/j.neuron.2014.05.014 24991964 PMC4082806

[B14] Davis MH, Coleman MR, Absalom AR, Rodd JM, Johnsrude IS, Matta BF, Owen AM, Menon DK (2007) Dissociating speech perception and comprehension at reduced levels of awareness. Proc Natl Acad Sci U S A 104:16032–16037. 10.1073/pnas.0701309104 17938125 PMC2042157

[B15] Fox MD, Raichle ME (2007) Spontaneous fluctuations in brain activity observed with functional magnetic resonance imaging. Nat Rev Neurosci 8:700–711. 10.1038/nrn2201 17704812

[B16] Fox MD, Snyder AZ, Vincent JL, Corbetta M, Van Essen DC, Raichle ME (2005) The human brain is intrinsically organized into dynamic, anticorrelated functional networks. Proc Natl Acad Sci U S A 102:9673–9678. 10.1073/pnas.0504136102 15976020 PMC1157105

[B17] Franks NP (2008) General anaesthesia: from molecular targets to neuronal pathways of sleep and arousal. Nat Rev Neurosci 9:370–386. 10.1038/nrn2372 18425091

[B18] Glerean E, Salmi J, Lahnakoski JM, Jääskeläinen IP, Sams M (2012) Functional magnetic resonance imaging phase synchronization as a measure of dynamic functional connectivity. Brain Connect 2:91–101. 10.1089/brain.2011.0068 22559794 PMC3624768

[B19] Glover GH, Li TQ, Ress D (2000) Image-based method for retrospective correction of physiological motion effects in fMRI: RETROICOR. Magn Reson Med 44:162–167. 10.1002/1522-2594(200007)44:1%3C162::AID-MRM23%3E3.0.CO;2-E 10893535

[B20] Gómez F, Phillips C, Soddu A, Boly M, Boveroux P, Vanhaudenhuyse A, Bruno MA, Gosseries O, Bonhomme V, Laureys S, Noirhomme Q (2013) Changes in effective connectivity by propofol sedation. PLoS One 8:e71370. 10.1371/journal.pone.0071370 23977030 PMC3747149

[B21] Guldenmund P, Demertzi A, Boveroux P, Boly M, Vanhaudenhuyse A, Bruno MA, Gosseries O, Noirhomme Q, Brichant JF, Bonhomme V, Laureys S, Soddu A (2013) Thalamus, brainstem and salience network connectivity changes during propofol-induced sedation and unconsciousness. Brain Connect 3:273–285. 10.1089/brain.2012.0117 23547875

[B22] Guldenmund P, Gantner IS, Baquero K, Das T, Demertzi A, Boveroux P, Bonhomme V, Vanhaudenhuyse A, Bruno MA, Gosseries O, Noirhomme Q, Kirsch M, Boly M, Owen AM, Laureys S, Gómez F, Soddu A (2016) Propofol-induced frontal cortex disconnection: a study of resting state networks, total brain connectivity, and mean BOLD signal oscillation frequencies. Brain Connect 6:225–237. 10.1089/brain.2015.0369 26650183

[B23] Hasson U, Yang E, Vallines I, Heeger DJ, Rubin N (2008) A hierarchy of temporal receptive windows in human cortex. J Neurosci 28:2539–2550. 10.1523/JNEUROSCI.5487-07.2008 18322098 PMC2556707

[B24] He BJ (2011) Scale-free properties of the functional magnetic resonance imaging signal during rest and task. J Neurosci 31:13786–13795. 10.1523/JNEUROSCI.2111-11.2011 21957241 PMC3197021

[B25] Hindriks R, Adhikari MH, Murayama Y, Ganzetti M, Mantini D, Logothetis NK, Deco G (2016) Can sliding-window correlations reveal dynamic functional connectivity in resting-state fMRI? Neuroimage 127:242–256. 10.1016/j.neuroimage.2015.11.055 26631813 PMC4758830

[B26] Honey CJ, Thesen T, Donner TH, Silbert LJ, Carlson CE, Devinsky O, Doyle WK, Rubin N, Heeger DJ, Hasson U (2012) Slow cortical dynamics and the accumulation of information over long timescales. Neuron 76:423–434. 10.1016/j.neuron.2012.08.011 23083743 PMC3517908

[B27] Huang Z, Dai R, Wu X, Yang Z, Liu D, Hu J, Gao L, Tang W, Mao Y, Jin Y, Wu X, Liu B, Zhang Y, Lu L, Laureys S, Weng X, Northoff G (2014) The self and its resting state in consciousness: an investigation of the vegetative state. Hum Brain Mapp 35:1997–2008. 10.1002/hbm.22308 23818102 PMC6868996

[B28] Huang Z, Zhang J, Wu J, Qin P, Wu X, Wang Z, Dai R, Li Y, Liang W, Mao Y, Yang Z, Zhang J, Wolff A, Northoff G (2016) Decoupled temporal variability and signal synchronization of spontaneous brain activity in loss of consciousness: an fMRI study in anesthesia. Neuroimage 124:693–703. 10.1016/j.neuroimage.2015.08.062 26343319

[B29] Huang Z, Zhang J, Longtin A, Dumont G, Duncan NW, Pokorny J, Qin P, Dai R, Ferri F, Weng X, Northoff G (2017) Is there a nonadditive interaction between spontaneous and evoked activity? Phase-dependence and its relation to the temporal structure of scale-free brain activity. Cereb Cortex 27:1037–1059. 10.1093/cercor/bhv288 26643354

[B30] Hubel DH (1988) Eye, brain, and vision. New York, NY: Scientific American Library.

[B31] Hudetz AG (2006) Suppressing consciousness: mechanisms of general anesthesia. Semin Anesth Perioper Med Pain 25:196–204. 10.1053/j.sane.2006.09.003

[B32] Hudetz AG (2012) General anesthesia and human brain connectivity. Brain Connect 2:291–302. 10.1089/brain.2012.0107 23153273 PMC3621592

[B33] Hudetz AG, Liu X, Pillay S (2015) Dynamic repertoire of intrinsic brain states is reduced in propofol-induced unconsciousness. Brain Connect 5:10–22. 10.1089/brain.2014.0230 24702200 PMC4313411

[B34] Huguenard JR, McCormick DA (2007) Thalamic synchrony and dynamic regulation of global forebrain oscillations. Trends Neurosci 30:350–356. 10.1016/j.tins.2007.05.007 17544519

[B35] Hutchison RM, Womelsdorf T, Allen EA, Bandettini PA, Calhoun VD, Corbetta M, Della Penna S, Duyn JH, Glover GH, Gonzalez-Castillo J, Handwerker DA, Keilholz S, Kiviniemi V, Leopold DA, de Pasquale F, Sporns O, Walter M, Chang C (2013) Dynamic functional connectivity: promise, issues, and interpretations. Neuroimage 80:360–378. 10.1016/j.neuroimage.2013.05.079 23707587 PMC3807588

[B36] Ishizawa Y, Ahmed OJ, Patel SR, Gale JT, Sierra-Mercado D, Brown EN, Eskandar EN (2016) Dynamics of propofol-induced loss of consciousness across primate neocortex. J Neurosci 36:7718–7726. 10.1523/JNEUROSCI.4577-15.2016 27445148 PMC4951576

[B37] Jordan D, Ilg R, Riedl V, Schorer A, Grimberg S, Neufang S, Omerovic A, Berger S, Untergehrer G, Preibisch C, Schulz E, Schuster T, Schröter M, Spoormaker V, Zimmer C, Hemmer B, Wohlschläger A, Kochs EF, Schneider G (2013) Simultaneous electroencephalographic and functional magnetic resonance imaging indicate impaired cortical top-down processing in association with anesthetic-induced unconsciousness. Anesthesiology 119:1031–1042. 10.1097/ALN.0b013e3182a7ca92 23969561

[B38] Kaneoke Y, Donishi T, Iwatani J, Ukai S, Shinosaki K, Terada M (2012) Variance and autocorrelation of the spontaneous slow brain activity. PLoS One 7:e38131. 10.1371/journal.pone.0038131 22666461 PMC3364220

[B39] Kondziella D, Friberg CK, Frokjaer VG, Fabricius M, Møller K (2016) Preserved consciousness in vegetative and minimal conscious states: systematic review and meta-analysis. J Neurol Neurosurg Psychiatry 87:485–492. 10.1136/jnnp-2015-310958 26139551

[B40] Ku SW, Lee U, Noh GJ, Jun IG, Mashour GA (2011) Preferential inhibition of frontal-to-parietal feedback connectivity is a neurophysiologic correlate of general anesthesia in surgical patients. PLoS One 6:e25155. 10.1371/journal.pone.0025155 21998638 PMC3187752

[B41] Laumann TO, Snyder AZ, Mitra A, Gordon EM, Gratton C, Adeyemo B, Gilmore AW, Nelson SM, Berg JJ, Greene DJ, McCarthy JE, Tagliazucchi E, Laufs H, Schlaggar BL, Dosenbach NUF, Petersen SE (2017) On the stability of BOLD fMRI correlations. Cereb Cortex 27:4719–4732. 10.1093/cercor/bhw265 27591147 PMC6248456

[B42] Laureys S, Schiff ND (2012) Coma and consciousness: paradigms (re)framed by neuroimaging. Neuroimage 61:478–491. 10.1016/j.neuroimage.2011.12.041 22227888

[B43] Lee U, Kim S, Noh GJ, Choi BM, Hwang E, Mashour GA (2009) The directionality and functional organization of frontoparietal connectivity during consciousness and anesthesia in humans. Conscious Cogn 18:1069–1078. 10.1016/j.concog.2009.04.004 19443244

[B44] Lee U, Ku S, Noh G, Baek S, Choi B, Mashour GA (2013) Disruption of frontal-parietal communication by ketamine, propofol, and sevoflurane. Anesthesiology 118:1264–1275. 10.1097/ALN.0b013e31829103f5 23695090 PMC4346246

[B45] Lerner Y, Honey CJ, Silbert LJ, Hasson U (2011) Topographic mapping of a hierarchy of temporal receptive windows using a narrated story. J Neurosci 31:2906–2915. 10.1523/JNEUROSCI.3684-10.2011 21414912 PMC3089381

[B46] Lewis LD, Weiner VS, Mukamel EA, Donoghue JA, Eskandar EN, Madsen JR, Anderson WS, Hochberg LR, Cash SS, Brown EN, Purdon PL (2012) Rapid fragmentation of neuronal networks at the onset of propofol-induced unconsciousness. Proc Natl Acad Sci U S A 109:E3377–E3386. 10.1073/pnas.1210907109 23129622 PMC3523833

[B47] Li D, Voss LJ, Sleigh JW, Li X (2013) Effects of volatile anesthetic agents on cerebral cortical synchronization in sheep. Anesthesiology 119:81–88. 10.1097/ALN.0b013e31828e894f 23508217

[B48] Lissek T, Obenhaus HA, Ditzel DA, Nagai T, Miyawaki A, Sprengel R, Hasan MT (2016) General anesthetic conditions induce network synchrony and disrupt sensory processing in the cortex. Front Cell Neurosci 10:64. 10.3389/fncel.2016.00064 27147963 PMC4830828

[B49] Liu X, Lauer KK, Ward BD, Li SJ, Hudetz AG (2013a) Differential effects of deep sedation with propofol on the specific and nonspecific thalamocortical systems: a functional magnetic resonance imaging study. Anesthesiology 118:59–69. 10.1097/ALN.0b013e318277a801 23221862 PMC4080838

[B50] Liu X, Pillay S, Li R, Vizuete JA, Pechman KR, Schmainda KM, Hudetz AG (2013b) Multiphasic modification of intrinsic functional connectivity of the rat brain during increasing levels of propofol. Neuroimage 83:581–592. 10.1016/j.neuroimage.2013.07.003 23851326 PMC3815996

[B51] Liu X, Lauer KK, Douglas Ward B, Roberts C, Liu S, Gollapudy S, Rohloff R, Gross W, Chen G, Xu Z, Binder JR, Li SJ, Hudetz AG (2017a) Propofol attenuates low-frequency fluctuations of resting-state fMRI BOLD signal in the anterior frontal cortex upon loss of consciousness. Neuroimage 147:295–301. 10.1016/j.neuroimage.2016.12.043 27993673 PMC5303656

[B52] Liu X, Lauer KK, Ward BD, Roberts CJ, Liu S, Gollapudy S, Rohloff R, Gross W, Xu Z, Chen G, Binder JR, Li SJ, Hudetz AG (2017b) Fine-grained parcellation of brain connectivity improves differentiation of states of consciousness during graded propofol sedation. Brain Connect 7:373–381. 10.1089/brain.2016.0477 28540741 PMC5685154

[B53] Marsh B, White M, Morton N, Kenny GN (1991) Pharmacokinetic model driven infusion of propofol in children. Br J Anaesth 67:41–48. 10.1093/bja/67.1.41 1859758

[B54] Martuzzi R, Ramani R, Qiu M, Rajeevan N, Constable RT (2010) Functional connectivity and alterations in baseline brain state in humans. Neuroimage 49:823–834. 10.1016/j.neuroimage.2009.07.028 19631277 PMC2764802

[B55] Mashour GA (2014) Top-down mechanisms of anesthetic-induced unconsciousness. Front Syst Neurosci 8:115. 10.3389/fnsys.2014.00115 25002838 PMC4066704

[B56] Meisel C, Bailey K, Achermann P, Plenz D (2017a) Decline of long-range temporal correlations in the human brain during sustained wakefulness. Sci Rep 7:11825. 10.1038/s41598-017-12140-w 28928479 PMC5605531

[B57] Meisel C, Klaus A, Vyazovskiy VV, Plenz D (2017b) The interplay between long- and short-range temporal correlations shapes cortex dynamics across vigilance states. J Neurosci 37:10114–10124. 10.1523/JNEUROSCI.0448-17.2017 28947577 PMC5647769

[B58] Murray JD, Bernacchia A, Freedman DJ, Romo R, Wallis JD, Cai X, Padoa-Schioppa C, Pasternak T, Seo H, Lee D, Wang XJ (2014) A hierarchy of intrinsic timescales across primate cortex. Nat Neurosci 17:1661–1663. 10.1038/nn.3862 25383900 PMC4241138

[B59] Ní Mhuircheartaigh R, Warnaby C, Rogers R, Jbabdi S, Tracey I (2013) Slow-wave activity saturation and thalamocortical isolation during propofol anesthesia in humans. Sci Transl Med 5:208ra148. 10.1126/scitranslmed.3006007 24154602

[B60] Northoff G, Huang Z (2017) How do the brain's time and space mediate consciousness and its different dimensions? temporo-spatial theory of consciousness (TTC). Neurosci Biobehav Rev 80:630–645. 10.1016/j.neubiorev.2017.07.013 28760626

[B61] Ponce-Alvarez A, Deco G, Hagmann P, Romani GL, Mantini D, Corbetta M (2015) Resting-state temporal synchronization networks emerge from connectivity topology and heterogeneity. PLoS Comput Biol 11:e1004100. 10.1371/journal.pcbi.1004100 25692996 PMC4333573

[B62] Power JD, Cohen AL, Nelson SM, Wig GS, Barnes KA, Church JA, Vogel AC, Laumann TO, Miezin FM, Schlaggar BL, Petersen SE (2011) Functional network organization of the human brain. Neuron 72:665–678. 10.1016/j.neuron.2011.09.006 22099467 PMC3222858

[B63] Power JD, Barnes KA, Snyder AZ, Schlaggar BL, Petersen SE (2012) Spurious but systematic correlations in functional connectivity MRI networks arise from subject motion. Neuroimage 59:2142–2154. 10.1016/j.neuroimage.2011.10.018 22019881 PMC3254728

[B64] Power JD, Mitra A, Laumann TO, Snyder AZ, Schlaggar BL, Petersen SE (2014) Methods to detect, characterize, and remove motion artifact in resting state fMRI. Neuroimage 84:320–341. 10.1016/j.neuroimage.2013.08.048 23994314 PMC3849338

[B65] Purdon PL, Pierce ET, Mukamel EA, Prerau MJ, Walsh JL, Wong KF, Salazar-Gómez AF, Harrell PG, Sampson AL, Cimenser A, Ching S, Kopell NJ, Tavares-Stoeckel C, Habeeb K, Merhar R, Brown EN (2013) Electroencephalogram signatures of loss and recovery of consciousness from propofol. Proc Natl Acad Sci U S A 110:E1142–E1151. 10.1073/pnas.1221180110 23487781 PMC3607036

[B66] Ranft A, Golkowski D, Kiel T, Riedl V, Kohl P, Rohrer G, Pientka J, Berger S, Thul A, Maurer M, Preibisch C, Zimmer C, Mashour GA, Kochs EF, Jordan D, Ilg R (2016) Neural correlates of sevoflurane-induced unconsciousness identified by simultaneous functional magnetic resonance imaging and electroencephalography. Anesthesiology 125:861–872. 10.1097/ALN.0000000000001322 27617689 PMC5069173

[B67] Scheffer M, Bascompte J, Brock WA, Brovkin V, Carpenter SR, Dakos V, Held H, van Nes EH, Rietkerk M, Sugihara G (2009) Early-warning signals for critical transitions. Nature 461:53–59. 10.1038/nature08227 19727193

[B68] Scheffer M, Carpenter SR, Lenton TM, Bascompte J, Brock W, Dakos V, van de Koppel J, van de Leemput IA, Levin SA, van Nes EH, Pascual M, Vandermeer J (2012) Anticipating critical transitions. Science 338:344–348. 10.1126/science.1225244 23087241

[B69] Schröter MS, Spoormaker VI, Schorer A, Wohlschläger A, Czisch M, Kochs EF, Zimmer C, Hemmer B, Schneider G, Jordan D, Ilg R (2012) Spatiotemporal reconfiguration of large-scale brain functional networks during propofol-induced loss of consciousness. J Neurosci 32:12832–12840. 10.1523/JNEUROSCI.6046-11.2012 22973006 PMC6703804

[B70] Schrouff J, Perlbarg V, Boly M, Marrelec G, Boveroux P, Vanhaudenhuyse A, Bruno MA, Laureys S, Phillips C, Pélégrini-Issac M, Maquet P, Benali H (2011) Brain functional integration decreases during propofol-induced loss of consciousness. Neuroimage 57:198–205. 10.1016/j.neuroimage.2011.04.020 21524704

[B71] Sepulcre J, Liu H, Talukdar T, Martincorena I, Yeo BT, Buckner RL (2010) The organization of local and distant functional connectivity in the human brain. PLoS Comput Biol 6:e1000808. 10.1371/journal.pcbi.1000808 20548945 PMC2883589

[B72] Sethi SS, Zerbi V, Wenderoth N, Fornito A, Fulcher BD (2017) Structural connectome topology relates to regional BOLD signal dynamics in the mouse brain. Chaos 27:047405. 10.1063/1.4979281 28456172

[B73] Shafer S (1996) STANPUMP User's Manual. Stanford University, Stanford, CA: Source. http://opentci.org/code/stanpump.

[B74] Stephens GJ, Honey CJ, Hasson U (2013) A place for time: the spatiotemporal structure of neural dynamics during natural audition. J Neurophysiol 110:2019–2026. 10.1152/jn.00268.2013 23926041 PMC3841928

[B75] Supp GG, Siegel M, Hipp JF, Engel AK (2011) Cortical hypersynchrony predicts breakdown of sensory processing during loss of consciousness. Curr Biol 21:1988–1993. 10.1016/j.cub.2011.10.017 22100063

[B76] Tagliazucchi E, Chialvo DR, Siniatchkin M, Amico E, Brichant JF, Bonhomme V, Noirhomme Q, Laufs H, Laureys S (2016) Large-scale signatures of unconsciousness are consistent with a departure from critical dynamics. J R Soc Interface 13:20151027. 10.1098/rsif.2015.1027 26819336 PMC4759808

[B77] Tononi G, Boly M, Massimini M, Koch C (2016) Integrated information theory: from consciousness to its physical substrate. Nat Rev Neurosci 17:450–461. 10.1038/nrn.2016.44 27225071

[B78] Uhrig L, Janssen D, Dehaene S, Jarraya B (2016) Cerebral responses to local and global auditory novelty under general anesthesia. Neuroimage 141:326–340. 10.1016/j.neuroimage.2016.08.004 27502046 PMC5635967

[B79] Van Dijk KR, Sabuncu MR, Buckner RL (2012) The influence of head motion on intrinsic functional connectivity MRI. Neuroimage 59:431–438. 10.1016/j.neuroimage.2011.07.044 21810475 PMC3683830

[B80] Wollstadt P, Sellers KK, Rudelt L, Priesemann V, Hutt A, Fröhlich F, Wibral M (2017) Breakdown of local information processing may underlie isoflurane anesthesia effects. PLoS Comput Biol 13:e1005511. 10.1371/journal.pcbi.1005511 28570661 PMC5453425

[B81] Wu T, Grandjean J, Bosshard SC, Rudin M, Reutens D, Jiang T (2017) Altered regional connectivity reflecting effects of different anaesthesia protocols in the mouse brain. Neuroimage 149:190–199. 10.1016/j.neuroimage.2017.01.074 28159688

[B82] Yan CG, Craddock RC, Zuo XN, Zang YF, Milham MP (2013) Standardizing the intrinsic brain: towards robust measurement of interindividual variation in 1000 functional connectomes. Neuroimage 80:246–262. 10.1016/j.neuroimage.2013.04.081 23631983 PMC4074397

[B83] Zang Y, Jiang T, Lu Y, He Y, Tian L (2004) Regional homogeneity approach to fMRI data analysis. Neuroimage 22:394–400. 10.1016/j.neuroimage.2003.12.030 15110032

[B84] Zang YF, He Y, Zhu CZ, Cao QJ, Sui MQ, Liang M, Tian LX, Jiang TZ, Wang YF (2007) Altered baseline brain activity in children with ADHD revealed by resting-state functional MRI. Brain Dev 29:83–91. 10.1016/j.braindev.2006.07.002 16919409

[B85] Zuo XN, Xu T, Jiang L, Yang Z, Cao XY, He Y, Zang YF, Castellanos FX, Milham MP (2013) Toward reliable characterization of functional homogeneity in the human brain: preprocessing, scan duration, imaging resolution and computational space. Neuroimage 65:374–386. 10.1016/j.neuroimage.2012.10.017 23085497 PMC3609711

